# The Respiratory Metabolism of *Polistes biglumis*, a Paper Wasp from Mountainous Regions

**DOI:** 10.3390/insects11030165

**Published:** 2020-03-04

**Authors:** Helmut Kovac, Helmut Käfer, Anton Stabentheiner

**Affiliations:** Institute of Biology, University of Graz, 8010 Graz, Austria; helmut.kaefer@uni-graz.at

**Keywords:** *Polistes biglumis*, respiratory metabolism, standard metabolic rate, activity, climate

## Abstract

European Polistine wasps inhabit mainly temperate and warm climate regions. However, the paper wasp *Polistes biglumis* represents an exception; it resides in mountainous areas, e.g., in the Alps and in the Apennines. In these habitats, the wasps are exposed to a broad temperature range during their lifetime. We investigated whether they developed adaptations in their metabolism to their special climate conditions by measuring their CO_2_ production. The standard or resting metabolic rate and the metabolism of active wasps was measured in the temperature range which they are exposed to in their habitat in summer. The standard metabolic rate increased in a typical exponential progression with ambient temperature, like in other wasps. The active metabolism also increased with temperature, but not in a simple exponential course. Some exceptionally high values were presumed to originate from endothermy. The simultaneous measurement of body temperature and metabolic rate revealed a strong correlation between these two parameters. The comparison of the standard metabolic rate of *Polistes biglumis* with that of *Polistes dominula* revealed a significantly lower metabolism of the alpine wasps. This energy saving metabolic strategy could be an adaptation to the harsh climate conditions, which restricts foraging flights and energy recruitment.

## 1. Introduction

The paper wasp *Polistes biglumis* resides mainly in mountainous areas, e.g., in the Alps and in the Apennines [1]. It is the only paper wasp that inhabits an alpine climate in Europe, and it is found at the boreal limit of Polistine wasps’ distribution in Japan [2]. The wasps live in typical rocky habitats with highly variable microclimate conditions [3]. The weather in spring is often rainy with low temperatures, but in summer it can be also quite warm. Therefore, the wasps are exposed to a broad temperature range during their lifetime, which is a challenge for the survival of the colonies. Due to the harsh climate conditions, the colony cycle is fairly short, beginning in April or May and ending, at the latest, in September, and the nests inhabited by the colonies are small and consist of a maximum of 30 wasps [1]. 

In (ectothermic) insects, the metabolism strongly depends on ambient temperature, see e.g., [4], and in Polistine and Vespine wasps it increases in an exponential course [5,6]. For modelling the energetic demand of a wasp colony, it is indispensable to know the wasps’ metabolism in relation to its dependence on ambient temperature. Especially, to assess the energetic demand and their chance of survival under future climate conditions, such investigations are of great importance.

We investigated the wasps’ standard metabolic rate (SMR) and the metabolism of active wasps in nearly the entire temperature range to which they are likely exposed to in their habitat during the breeding season. The standard metabolic rate, usually equated with resting metabolism, is a very important parameter in an insect’s life, representing the energetic costs of simple subsistence. It determines an individual’s minimum energy requirement under a standardized set of conditions, and is a fundamental parameter for comparing the relative energy expenditures of particular activities. As Polistine wasps spend a lot of time (more than 50%, personal observations) resting at their nest, the SMR is an important parameter in their energy balance. Additionally, the metabolism during activity (walking, grooming) was also determined, in order to assess the energetic demand of activity in comparison to the SMR. As Polistine wasps are capable of endothermic heat production, see e.g., [7,8], which requires additional energy, we conducted special experiments to measure the metabolism in the endothermic state and to evaluate the relation between endothermic heat production and metabolic rate. Furthermore, we determined the critical thermal maximum (CT_max_), the point where coordinated activity ceases.

The metabolic cold adaptation theory predicts that populations or species from cooler environments should have either a higher metabolic rate at a certain temperature or a steeper relationship of respiratory metabolism on temperature (a greater sensitivity to temperature), see e.g., [9,10,11,12]. However, the general validity of this hypothesis is still contentious [10]. Special metabolic adaptations may be necessary to survive in habitats with harsh conditions. These adaptations may, for example, be an increase of the energy metabolism to support activity at low temperatures [9,10,11,12]. However, there is a considerable amount of reports of animals, which do not react in this way, or did not evolve in this direction [12]. An alternative strategy to be successful in a cold environment is to reduce the overall energy demand, either by simple behavioral means like prolonged resting phases or movement to cooler microhabitats, or by reduction of the energy metabolism. In social insects like Polistine wasps, which must stay on the nest to protect their brood, metabolic adaptations have to be considered. In order to reveal environmental adaptations, we included data of *Polistes dominula* from a study by Käfer et al. [5], a species from a different habitat and microclimate. In contrast to *Polistes biglumis*, *Polistes dominula* is a species with a very large distribution range in different climate regions. The aim of this study, therefore, was to investigate, whether *P. biglumis* has developed a special mode of adaptation of their metabolism in response to the extreme environmental conditions in their habitat.

## 2. Materials and Methods 

### 2.1. Animals

A first series of experiments on resting and active metabolism was conducted in August 2018. Workers of *Polistes biglumis* were collected from seven nests at a natural site at Krakauhintermühlen (47°11′20.29″ N, 13°57′22.03″ E; 1320 m ASL) in Styria, Austria. During the time of experiments, which lasted about two weeks, the wasps were kept in small cages at room temperature (~21–25 °C) and were provided with honey. A second series of experiments on the wasps’ active metabolism and endothermic heat production was conducted in August 2019, where workers of *Polistes biglumis* from three nests at Teichalm (47°21′19.24″ N, 15°26′38.11″ E; 1170 m ASL) in Styria, Austria, were collected and kept similar like in 2018.

### 2.2. Experimental Set-Up and Measurement Procedures

**Figure 1:**
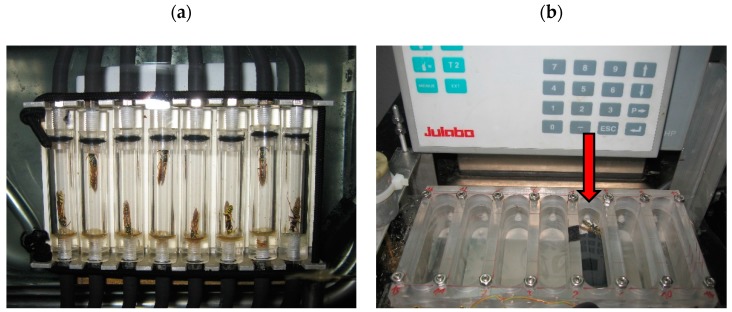
(**a**) Eight measurement chambers to measure the CO_2_ production rate of active and resting wasps (*Polistes biglumis*). (**b**) Seven measurement chambers in the water bath to measure the endothermic activity and CO_2_ production rate of active and resting wasps (*Polistes biglumis*). The arrow indicates the stocked measurement chamber.

Prior to the experiments, the wasps were weighed with an accuracy of 0.1 mg (Schimadzu AUW-120DV, Nishinokyo Kuwabara-cho, Nakagyo-ku, Kyoto, Japan). Then, the wasps were put in small plastic tubes (length 35 mm, diameter 9 mm, volume 2.23 mL), which served as measurement chambers (Figure 1a). Eight of these tubes were connected with an eight-channel multiplexer (RM Gas Flow Multiplexer, Sable Systems, Las Vegas, Nevada, USA), which controlled the flushing and closing of the chambers and enabled the serial measurement. The chambers were submerged in a water bath (Julabo F33, JULABO Labortechnik GmbH, Seelbach, Germany) for temperature control with an accuracy of 0.1 °C. Eight wasps were tested per day at only one experimental temperature. The experiments were conducted in a temperature range of 5 to 45 °C, in steps of 5 °C. Trials with an experimental temperature of 15, 25, and 35 °C were conducted two times, the others at 5, 10, 20, 30, and 40 °C, only at one time. After a habituation time of 15 min, the light was turned off and the wasps were left in the dark in order to get resting phases. One experiment lasted four hours with the exception of the 5 °C trial, which lasted 13 h to accumulate enough CO_2_ in the chambers for an accurate measurement.

A video camera was installed to monitor the wasps’ activity and to assess the behavior during later evaluation (Sony GDR-CX730E, Sony Europe Limited, Vienna, Austria). For further data analysis, we distinguished between resting and active behavior (mainly walking and grooming).

In these experiments the carbon dioxide emission from the wasps’ respiratory metabolism (CO_2_ production) was measured. It is commonly used as an indirect measure of an organism’s metabolic rate. The respirometry measurements were conducted in “stop-and-go” mode. For this purpose, the multiplexer with the measurement chambers was connected to a differential infrared gas analyser (DIRGA; URAS 14, ABB, Zürich, Switzerland). The insects’ CO_2_ release was measured with an accuracy of ~2 ppm. To maximize the system sensitivity (<0.2 ppm), the air was taken from outside the laboratory. Before it entered the reference tube of the DIRGA, the air was pumped through a 10 l container to dampen fluctuations in CO_2_ content, passed the pump and mass flow controllers (0–1000 mL min^−1^, Brooks 5850 S), and then passed through another container (5 l) for additional CO_2_ and pressure fluctuation damping. To maintain the relative humidity in the measurement chambers at about 50%, the air was humidified by passing it through two bottles filled with distilled water. The air entering these water bottles had to passage a “bubbler” to produce air bubbles. Before the air entered the URAS reference and measurement tubes (where it was heated to 60 °C), it was dried by passing it through two Peltier-driven cool traps (10 °C). The airflow in the system was 144 mL min^−1^. The volumes (nl) of CO_2_ production reported in this paper refer to standard (STPS) conditions (0 °C, 101.32 kPa = 760 Torr). The CO_2_ release was recorded at one-second intervals. At the beginning and at the end of each experimental run the gas analyser was calibrated automatically in zero and end point by the use of internal calibration cuvettes, and the data were corrected for any remaining drift or offset. For further methodical details see [13].

In a second series of experiments in 2019, we investigated the relation between metabolism and endothermic heat production by measuring the CO_2_ production and the body temperature simultaneously. Single wasps were placed in a self-constructed plastic measurement chamber (Figure 1b). The inner dimension of the measurement chamber was 74 × 13 × 23 mm, which results in a volume of 26 mL. The upper part of the chamber was closed by a thin cellophane film, which allowed the measurement of the wasps’ body surface temperature by infrared thermography. The lower part of the chamber was submerged in a water bath (Julabo F33, JULABO Labortechnik GmbH, Seelbach, Germany) for temperature control. For the measurement of the wasps’ CO_2_ production, the chamber was connected to the differential infrared gas analyser as described above. The ambient temperature in the chamber was controlled by an inserted thermocouple (NiCr/Ni) connected to a data logger (ALMEMO 2890-9, Ahlborn GmbH, Holzkirchen, Germany). To evoke high activity and an endothermic reaction in the wasps, we shook the chamber when the wasps were calm. After ten minutes, where we had tried to provoke high activity, we turned off the light and left the wasps alone to get data for low activity or resting phases. One trial lasted 20 to 30 min, and then another wasp was tested. The experiments were conducted with sixteen wasps at a temperature range of 23–31 °C.

**Figure 2:**
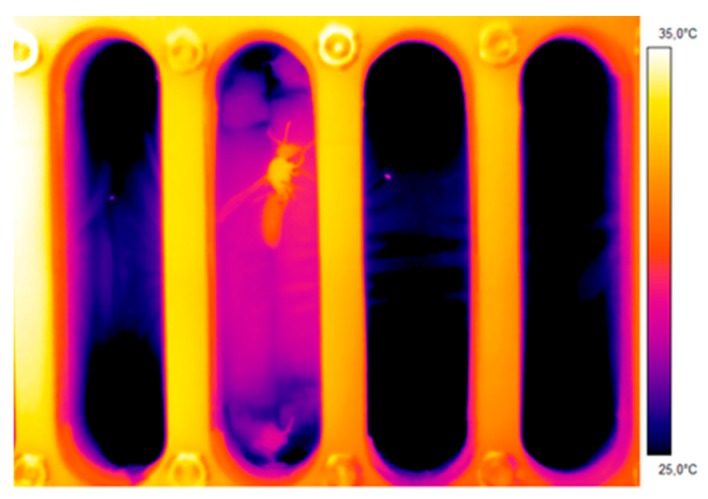
Thermogram of a wasp (*Polistes biglumis*) in a measurement chamber to measure the endothermic activity and CO_2_ production rate.

The surface temperature of the wasps’ body (head, thorax, abdomen) was measured by infrared thermography at a frame rate of 30 Hz (T650sc, FLIR Systems Inc., Danderyd, Sweden). The measured body temperature was calibrated to ~0.5 °C accuracy, assuming a wasp cuticle infrared emissivity of 0.97 [14,15] and using a proprietary Peltier-driven reference source of known temperature and emissivity for camera calibration. For details see [13,14,15,16]. Infrared data collection was done in real-time and stored digitally on an internal memory card or externally at a personal computer’s hard drive, and evaluated later in the laboratory. Evaluation of the surface temperatures of head (T_head_), thorax (T_thorax_), and abdomen (T_abdomen_) (Figure 2) was done with AGEMA Research software (FLIR Systems Inc., Wilsonville, USA) controlled by a proprietary Excel (Microsoft Corporation, Redmond, USA) VBA macro. Activity and behavior were also evaluated from the infrared video recordings.

To relate the respiration measurements to the thermographic measurements, the evaluated CO_2_ production rate (VCO_2_, nL s^−1^) and the thorax temperature excess (T_thorax_–T_abdomen_) were plotted in dependence on time, compensated for the delay of VCO_2_ recordings relative to the thermographic measurements (Figure 5). Then we averaged the CO_2_ production rate and the thorax temperature excess at phases of high activity (mainly walking) and low activity (grooming or resting), which lasted one to four minutes, and correlated these two parameters (Figure 5). This data evaluation was conducted separately for experiments at mean experimental temperatures (±SD) of 25.7 ± 0.7 °C and 29.9 ± 0.5 °C.

### 2.3. Critical Thermal Maximum (CT_max_)

To determine the upper limit of activity, we conducted a so-called “critical thermal maximum” experiment (activity CT_max_) [17]. In two trials, seven wasps were individually placed in the plastic tubes as described above (in the first series of experiments) and put in the water bath. The temperature of the water bath was increased from 25 °C to 55 °C at a dT = 0.25 °C min^−1^. The point of time when controlled motoric activity ceased and muscle spasms started was determined via behavioral observation, and the temperature at that time (CT_max_) was extracted from the logger file of a thermocouple, which was recording the temperature inside the measurement chamber. For further information see [13,18].

### 2.4. Microclimate Measurements

**Figure 3:**
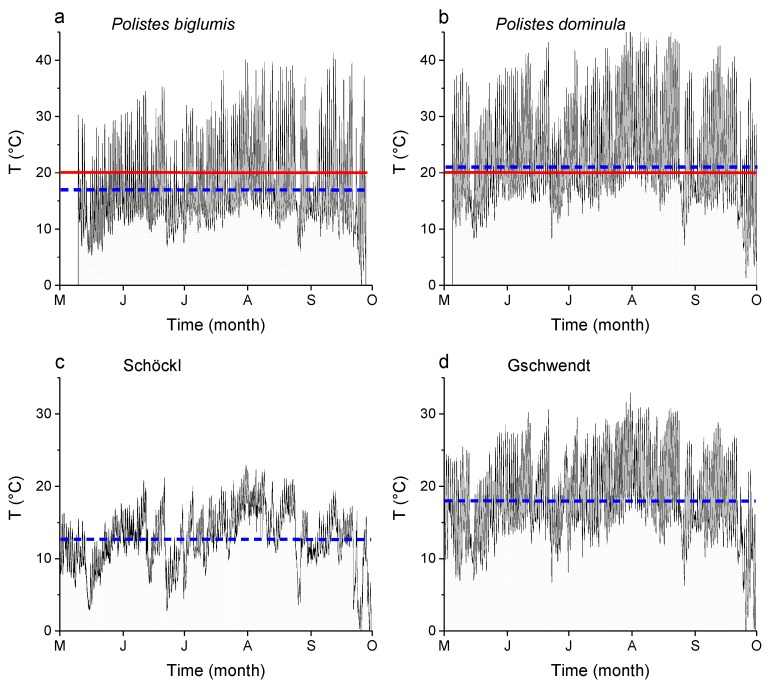
Temperature recordings of the microclimate at a nest of (**a**) *Polistes biglumis* and (**b**) *Polistes dominula*, and standard recordings of the ambient temperature at the nearest weather station to the *Polistes biglumis* nest at (**c**) Schöckl (47°11′53.25″ N, 15°27′56.18″ E; 1445 m ASL) and to the *Polistes dominula* nest at (**d**) Gschwendt (47°10′41.62″ N, 15°34′22.52″ E; 522 m ASL). The blue line (dashed) indicates the mean of the recorded period from May to October 2018, and the red line (solid) marks the threshold of onset of foraging activity.

To measure the microclimate during a breeding season, from May to October 2018 data loggers were installed at a nest of *Polistes biglumis*, and, for comparison at a nest of *Polistes dominula*. The temperature at the nest of *Polistes biglumis* was measured in an open forest area at Teichalm (47°21′19.24″ N, 15°26′38.11″ E) in Styria, Austria with an MSR data logger (MSR Electronics GmbH, Seuzach, Switzerland). The temperature at the nest of *Polistes dominula* was measured at a loft in Gschwendt (47°10′41.62″ N, 15°34′22.52″ E) in Styria, Austria with an Ahlborn data logger (ALMEMO 2290-8, Ahlborn GmbH, Holzkirchen, Germany). To characterize the climate in the region, we present the ambient temperature recordings from May to October 2018 of a public weather station at the Schöckl mountain (ZAMG—Zentralanstalt für Meteorologie und Geodynamik, Vienna, Austria; 47°11′53.25″ N, 15°27′56.18″ E; 1445 m ASL; 15 km from the nests of *Polistes biglumis*), which is the nearest one to Teichalm, and of an own weather station of the University, in Gschwendt (47°10′41.62″ N, 15°34′22.52″ E; 522 m ASL) (Figure 3). This weather station was in the close vicinity (50 m) to the nests of *Polistes dominula*.

### 2.5. Data Analysis

All calculations were done with MS Excel (Microsoft Corporation, Redmond, WA, USA) and with Origin 2017 software (OriginLab, OriginLab Corporation, Northampton, MA, USA). Curve fitting was done with Origin. The average values for the evaluated parameters mentioned in the results derive from the curve fitting. Statistics were done with Statgraphics software (Statgraphics Centurion XVI, StatPoint Technology Inc., The Plains, VA, USA). First, “general linear model (GLM)” statistics was performed to test the influence of ambient temperature and species on the measured and calculated parameters. Furthermore, simple linear regressions in combination with an ANOVA were performed to test the dependence of the metabolic rate on ambient temperature and to compare between activity or resting metabolic rate or species specific metabolic rate.

## 3. Results

### 3.1. Standard Metabolic Rate (SMR) and Activity Metabolic Rate (AMR)

**Figure 4:**
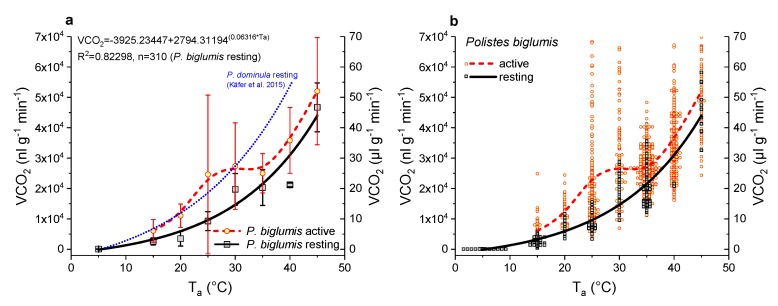
(**a**) Means ± SD and (**b**) single values of CO_2_ production rate in dependence on ambient temperature (T_a_) of active (red) and resting (black) wasps (*Polistes biglumis*). The dotted line (blue) represents the CO_2_ production rate of *Polistes dominula* from Käfer et al. [5].

The mean weight of all investigated wasps in these experiments was 65.4 ± 15.1 mg (n = 87). The SMR of these wasps increased with ambient temperature in a typical exponential course (Figure 4). The lowest mean value of VCO_2_ (derived from the curve fitting in Figure 4) at an ambient temperature of 5 °C was 0.14 µL g^−1^ min^−1^. The VCO_2_ increased to 3.29 µL g^−1^ min^−1^ at 15 °C, to 9.65 µL g^−1^ min^−1^ at 25 °C, to 21.61 µL g^−1^ min^−1^ at 35 °C, and reached 44.01 µL g^−1^ min^−1^ at 45 °C.

The AMR of wasps, which were mainly walking or grooming, could not be described by a simple exponential function (Figure 4), so we used a B-spline to connect the data points. It was always higher than the SMR (*p* < 0.001, ANOVA). Below 15 °C, we could not observe any activity, the wasps remained calm after insertion into the measurement chamber. At 15 °C, the VCO_2_ of the active wasps was 6.08 µL g^−1^ min^−1^. It increased to 24.63 µL g^−1^ min^−1^ at 25 °C and remained approximately at this level at 30 and 35 °C. At higher temperatures, the VCO_2_ increased further and reached 52.03 µL g^−1^ min^−1^ at 45 °C.

### 3.2. Endothermic Performance and Metabolic Rate

In these experiments, the mean weight of the wasps was 66.2 ± 16.9 mg (n = 16). The endothermic heat production of the wasps’ thoracic muscles is clearly visible in Figure 2. As a measure for the wasps’ degree of endothermy, we chose the difference between the temperature of the thorax and the abdomen, the so-called thorax temperature excess (T_thorax_ − T_abdomen_). Despite heavy stimulation the degree of endothermy remained small. The maximum thorax temperature excess at a mean experimental temperature of 25.7 °C was 2.7 °C (n = 691 evaluated thermograms). At a mean experimental temperature of 29.9 °C, it was 2.9 °C (n = 1148).

**Figure 5:**
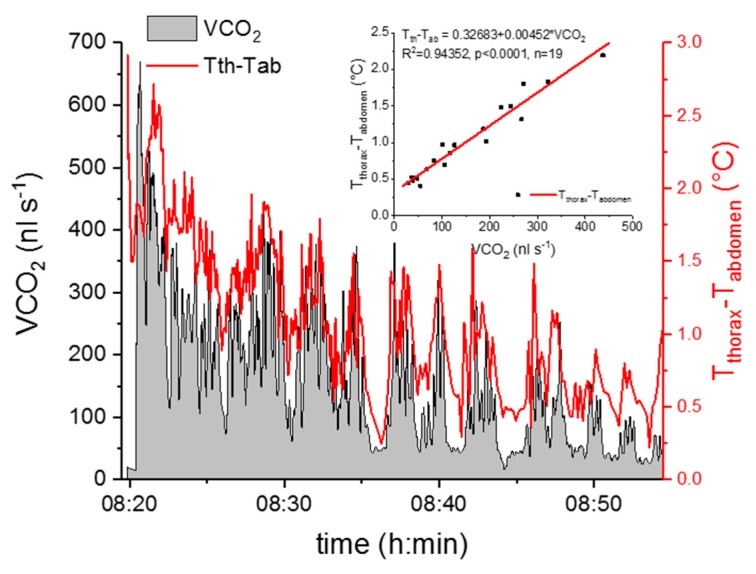
The CO_2_ production rate and the thorax temperature excess (T_thorax_–T_abdomen_) of an active wasp (*Polistes biglumis*) in dependence on time. The insert shows the correlation between the CO_2_ production rate and the thorax temperature excess.

**Figure 6:**
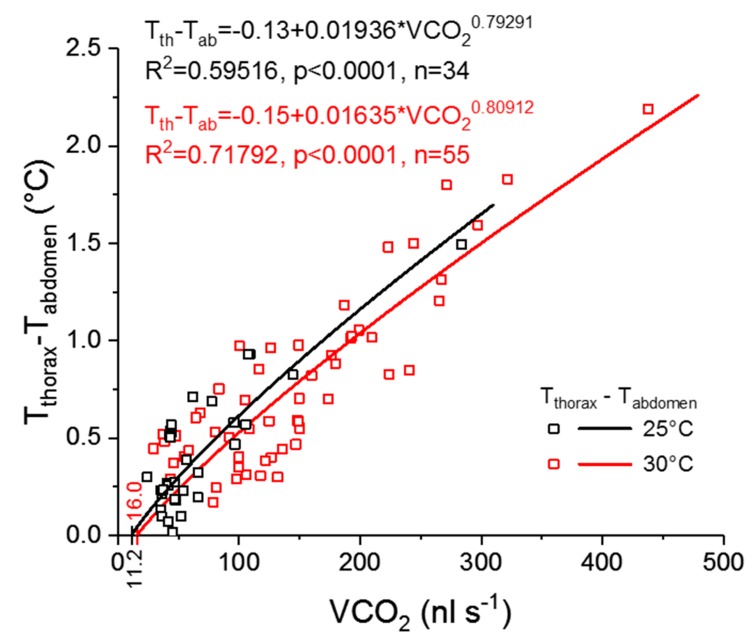
The correlation between the CO_2_ production rate and the thorax temperature excess (T_thorax_–T_abdomen_) of active wasps (*Polistes biglumis*) at an experimental temperature of 25 °C (black) and 30 °C (red). Indicated are the mean threshold limit values of CO_2_ production rate, 11.2 nL s^−1^ at 25 °C and 16.0 nL s^–1^ at 30 °C, derived from the SMR curve-fitting (Figure 4). At these values, the thorax temperature excess is assumed to be (close to) zero.

A strong positive correlation between the wasps’ thorax temperature excess and the CO_2_ production rate could be detected (Figure 5 and Figure 6). Figure 5 represents one example of an experiment. The simultaneously measured thorax temperature excess and the CO_2_ production rate are plotted in dependence on time, and the insert shows the correlation between the two parameters. The thorax temperature excess increased strongly with an increasing CO_2_ production rate. In Figure 6, the results of all investigated wasps for the two experimental temperatures are presented and reveal the strong correlation of the two parameters at both conditions (*p* < 0.0001). From the curve fitting of the SMR (Figure 4), we extracted the threshold limit values for the resting metabolism at 25 °C (11.2 nL s^−1^) and 30 °C (16.0 nL s^−1^). As resting wasps should exhibit no (or only weak) endothermic activity, we therefore assumed the temperature excess to be zero (or close to zero) at these values. With this assumption, we calculated the curve fitting for the two temperature conditions with an allometric function. Both curves show a very similar course, with an almost linear increase of the thorax temperature excess at higher CO_2_ emission rates (Figure 6).

### 3.3. The Critical Thermal Maximum (CT_max_)

The upper limit of coordinated movement or activity CT_max_ determined in 14 wasps (mean weight 70.6 ± 13.0 mg) was observed at 47.2 ± 0.5 °C.

## 4. Discussion

Temperature is one of the most important abiotic factors determining an insect’s energy balance. In this study, the standard metabolic rate (SMR) of *Polistes biglumis* was found to increase strongly in an exponential course with ambient temperature as expected (Figure 4). A similar correlation between SMR and temperature was also detected in other Polistine and Vespine wasps, e.g., *Polistes dominula* [5] and *Vespula vulgaris* [6]. It is the underlying basic biochemical and physiological processes, which determine an insect’s minimum energy demand. These processes are considered similar in related species [19]. However, regarding the thermal sensitivity of insect metabolism (Q_10_), i.e., the influence of ambient temperature on it, there nevertheless may occur differences in the energy demand of closely related species or even subspecies, living in different habitats, see e.g., [9,10,20,21,22]. Indeed, we could detect such differences, when we compared the SMR of *Polistes biglumis* with that of *Polistes dominula* from the study by Käfer et al. [5]. With the exception at low temperatures (5 °C), the SMR of *Polistes biglumis* was always considerably below that of *Polistes dominula* and differed significantly (Figure 4; *p* << 0.0001, ANOVA). The covariate ‘weight’ had no significant effect (*p* = 0.11). The metabolic rate of *Polistes dominula* is about 73%, 87%, and 100% higher than that of *Polistes biglumis* (at T_a_ = 40 °C, 30 °C, and 20 °C, respectively). The smaller size of *P. biglumis* cannot account for its lower metabolism, because smaller insects tend to have a higher mass-specific metabolic rate [6,23,24]. This means a great difference in the energy expenditure during the resting phases of the wasps. As Polistine wasps spend a lot of time motionless at their nests (more than 50% in *P. dominula* and *P. biglumis*, personal observations), these phases make up a not negligible amount of their energy demand. The question arises: Why there are such differences in the SMR of these quite similar paper wasps? An explanation could be found in the different habitats and climates the two species inhabit. *Polistes dominula* is an originally Mediterranean species, which settles now mainly in the lowlands of the Mediterranean and the temperate climate regions. European *Polistes biglumis*, on the other hand, inhabits mainly mountainous regions in the Apennines and the Alps, with a harsher alpine climate. Foraging in Polistine wasps depends strongly on ambient temperatures [25]. *Polistes biglumis* start foraging activity only at temperatures above 20 °C (authors’ unpublished observations). In the mountainous climate, the foraging activity is more restricted due to more frequent clouds or rainy weather conditions and generally lower temperatures. We could demonstrate this with temperature recordings of the microclimate at nests of both wasp species (Figure 3). The recordings showed that the mean temperature during the breeding season in 2018 was clearly lower at a *Polistes biglumis* nest (16.9 °C versus 21.0 °C), and also the mean temperature at the nearest weather station was clearly lower in the mountainous region (12.6 °C versus 18.0 °C). In addition, from the temperature curves we can conclude that the frequency of temperatures higher than 20 °C was lower in the *Polistes biglumis* habitat. This means that *Polistes biglumis* has less time with suitable temperatures for foraging flights available. Therefore, the wasps have to be more economical with their energy resources, and have developed towards a lower SMR as an adaptation to a harsher environment than comparable related species from warmer habitats. How this is achieved is still unknown. Beside other pathways of energy metabolism, both a decrease in mitochondrial density or mitochondrial aerobic capacity have to be taken into account [12].

What does this finding mean in the context of an increasing temperature due to climate change? On the first hand, an increasing temperature means an increasing energy demand for the SMR. On the other hand, it could also mean more favourable temperature conditions for foraging. However, we cannot foresee whether there will be more prey available or that the wasps will be able to catch more prey. Therefore, forecasting whether the wasps will benefit or suffer from higher temperatures is not simple and requires further research. Regardless, wasps are looking at an uncertain future and should be adapted to unpredictable weather.

The metabolic cold adaptation theory predicts that populations or species from cooler environments should have either a higher metabolic rate at a certain temperature, or a steeper relationship of respiratory metabolism with temperature (a greater sensitivity to temperature), see e.g., [9,10,21,22]. However, as already stated by Terblanche et al. [10], the generality of this hypothesis is contentious. While there is evidence that this may occur in aquatic [12] and terrestrial poikilothermic animals [9,10,11], May et al. [22] could not find support in a comparison of northern and southern populations of gypsy moths (*Lymantria dispar*) introduced to America. Our comparison, between *P. biglumis* from colder mountainous habitats showing a considerably lower metabolic rate and sensitivity to temperature than *P. dominula* from the warmer lowlands (Figure 4), seems to call the generality of the metabolic cold adaptation theory in question. These two related species [26] may have had ample time to adapt to their specific environments. As was already mentioned, *P. biglumis* may have been forced to develop an even more energy-extensive living style than *P. dominula*, i.e., to save energy whenever possible. In conclusion, we think that one should consider the metabolic cold adaptation theory as a possibility of reaction or adaptation, and not so much as a general necessity.

Terblanche et al. [10] pointed out the necessity to use only true resting (standard) metabolic curves in such comparisons. Otherwise, the metabolic rate to temperature relationship may be considerably different and variable. Therefore, we followed a strict protocol to separate measurements of resting from those of active individuals. As we had expected, the metabolism of active wasps was always higher than their resting metabolism, especially at 25 °C and 30 °C (Figure 4). Locomotion is always accompanied by additional energy demand, see e.g., [27,28,29,30,31,32]. In *Polistes dominula*, the energy demand of all non-resting behaviors (walking, buzz-walking, and interaction behavior) were at least twice as high as the resting rate, with buzz-walking having the highest rate [32]. The rate of energy use in buzz-walking (a combination of rapid walking and very brief airborne intervals) averaged 7.1 times that of rest. In our investigation, the highest mean of active metabolism (of walking wasps) was on average 2.7 times that of the resting metabolic rate (at T_a_ = 25 °C, Figure 4). However, the variability (standard deviation) was enormous. Therefore, we presumed that there could be a difference in the wasps’ endothermic state, and we conducted additional experiments. The simultaneous measurement of the body temperature and the metabolic rate of the active wasps confirmed this and revealed a strong positive correlation between the two parameters (Figure 5 and Figure 6). However, endothermy by activation of thoracic flight muscles, and thus the increase of metabolism, remained moderate despite heavy stimulation of the wasps (Figure 2, Figure 5 and Figure 6). This underpins the energy-saving nature of these paper wasps. In foraging Vespine wasps [33,34] and honeybees [35,36], this relationship between the endothermic performance and metabolism not only extends to much higher values, but they can (and do) also maintain endothermy for much longer periods. In these strongly endothermic insects, however, the relationship is much more complex [34,36,37,38].

In contrast to the SMR, the upper thermal limit or CT_max_ of activity we determined for *Polistes biglumis* (CT_max_ = 47.2 °C) was virtually identical to that of *Polistes dominula* (CT_max_ = 47.1 °C) [18]. This is a surprising result, as one would expect that the species living in a warmer habitat has been endowed with a higher thermal tolerance (upper thermal limit), as was determined in seed bug species [39]. Therefore, we suggest that the lower metabolism and thermal sensitivity in *P. biglumis* is not a result of a simple shift of the range of thermal tolerance (CT_max_ − CT_min_) to higher temperatures, as would be expected by a shift of the whole metabolic curve to higher temperatures (as might be expected by generally higher thermal optima of the involved enzymes).

## 5. Conclusions

The SMR of *Polistes biglumis* is significantly lower than that of *Polistes dominula*. We suggest its economizing lifestyle to be an adaptation to the harsher climate conditions in its mountainous habitat.

## References

[1] Fucini S., Di Bona V., Mola F., Piccaluga C., Lorenzi M. (2009). Social wasps without workers: Geographic variation of caste expression in the paper wasp *Polistes biglumis*. Insect. Soc..

[2] Yamane S., Kawamichi T. (1975). Bionomic Comparison of *Polistes biglumis*. Kontyû Tokyo.

[3] Lorenzi M.C., Turillazzi S. (1986). Behavioral and ecological adaptations to the high mountain environment of *Polistes biglumus bimaculatus*. Ecol. Entomol..

[4] Chown S.L., Nicolson S.W. (2004). Insect Physiological Ecology: Mechanisms and Patterns.

[5] Käfer H., Kovac H., Oswald B., Stabentheiner A. (2015). Respiration and metabolism of the resting European paper wasp (*Polistes dominulus*). J. Comp. Physiol. B.

[6] Käfer H., Kovac H., Stabentheiner A. (2012). Resting metabolism and critical thermal maxima of vespine wasps (*Vespula* sp.). J. Insect. Physiol..

[7] Kovac H., Stabentheiner A., Schmaranzer S. (2009). Thermoregulation of water foraging wasps (*Vespula vulgaris* and *Polistes dominulus*). J. Insect. Physiol..

[8] Weiner S.A., Upton C.T., Noble K., Woods W.A., Starks P.T. (2010). Thermoregulation in the primitively eusocial paper wasp, *Polistes dominulus*. Insect. Soc..

[9] Addo-Bediako A., Chown S.L., Gaston K.J. (2002). Metabolic cold adaptation in insects: A large-scale perspective. Funct. Ecol..

[10] Terblanche J.S., Clusella-Trullas S., Deere J.A., Van Vuuren B.J., Chown S.L. (2009). Directional evolution of the slope of the metabolic rate-temperature relationship is correlated with climate. Physiol. Biochem. Zool..

[11] Chown S.L., Haupt T.M., Sinclair B.J. (2016). Similar metabolic rate-temperature relationships after acclimation at constant and fluctuating temperatures in caterpillars of a sub-Antarctic moth. J. Insect. Physiol..

[12] Pörtner H.O. (2002). Climate variations and the physiological basis of temperature dependent biogeography systemic to molecular hierarchy of thermal tolerance in animals. Comp. Biochem. Physiol. A.

[13] Stabentheiner A., Kovac H., Hetz S.K., Käfer H., Stabentheiner G. (2012). Assessing honeybee and wasp thermoregulation and energetics—New insights by combination of flow-through respirometry with infrared thermography. Thermochim. Acta.

[14] Kovac H., Stabentheiner A. (1999). Efect of food quality on the body temperature of wasps (*Paravespula vulgaris*). J. Insect. Physiol..

[15] Stabentheiner A., Schmaranzer S. (1987). Thermographic determination of body temperatures in honey bees and hornets: Calibration and applications. Thermology.

[16] Schmaranzer S., Stabentheiner A. (1988). Variability of the thermal behavior of honeybees on a feeding place. J. Comp. Physiol. B.

[17] Lighton J.R.B., Turner R.J. (2004). Thermolimit respirometry: An objective assessment of critical thermal maxima in two sympatric desert harvester ants. Pogonomyrmex rugosus and P. californicus. J. Exp. Biol..

[18] Kovac H., Käfer H., Petrocelli I., Stabentheiner A. (2017). Comparison of thermal traits of *Polistes dominula* and *Polistes gallicus*, two European paper wasps with strongly differing distribution ranges. J. Comp. Physiol. B.

[19] Lake S.L., MacMillan H.A., Williams C.M., Sinclair J.B. (2013). Static and dynamic approaches yield similar estimates of the thermal sensitivity of insect metabolism. J. Insect. Physiol..

[20] Fangue N.A., Richards J.G., Schulte P.M. (2009). Do mitochondrial properties explain intraspecific variation in thermal tolerance. J. Exp. Biol..

[21] Vorhees A.S., Gray E.M.J., Bradley J.T. (2013). Thermal resistance and performance correlate with climate in populations of a widespread mosquito. Physiol. Biochem. Zool..

[22] May C., Hillerbrand N., Thompson L.M., Faske T.M., Martinez E., Parry D., Agosta S.J., Grayson K.L. (2018). Geographic Variation in Larval Metabolic Rate Between Northern and Southern Populations of the Invasive Gypsy Moth. J. Ins. Sci..

[23] Vogt J.T., Appel A.G. (1999). Standard metabolic rate of the fire ant, *Solenopsis invicta* Buren: Effects of temperature, mass, and caste. J. Insect. Physiol..

[24] Niven J.E., Scharlemann J.P.W. (2005). Do insect metabolic rates at rest and during flight scale with body mass?. Biol. Let..

[25] Nannoni A., Cervo R., Turillazzi S. (2001). Foraging activity in European Polistes wasps (Hymenoptera, Vespidae). Boll. Soc. Entomol. Italiana.

[26] Schmid-Egger C., van Achterberg K., Neumeyer R., Morinière J., Schmidt S. (2017). Revision of the West Palaearctic Polistes Latreille, with the description of two species–an integrative approach using morphology and DNA barcodes (Hymenoptera, Vespidae). ZooKeys.

[27] Bartholomew G.A., Lighton J.R.B., Louw G.N. (1985). Energetics of locomotion and patterns of respiration in tenebrionid beetles from the Namib Desert. J. Comp. Physiol. B.

[28] Lighton J.R.B., Feener D.H. (1989). A comparison of energetics and ventilation of desert ants during voluntary and forced locomotion. Nature.

[29] Berrigan D., Lighton J.R.B. (1994). Energetics of pedestrian locomotion in adult male blowflies, *Protophormia terraenovae* (Diptera: Calliphoridae). Physiol. Zool..

[30] Duncan F.D., Lighton J.R.B. (1997). Discontinuous ventilation and energetics of locomotion in the desert-dwelling female mutillid wasp, *Dasymutilla gloriosa*. Physiol. Entomol..

[31] Lipp A., Wolf H., Lehmann F.O. (2005). Walking on inclines: Energetics of locomotion in the ant *Camponotus*. J. Exp. Biol..

[32] Weiner S.A., Woods W.A., Starks P.T. (2009). The energetic costs of stereotyped behavior in the paper wasp, *Polistes dominulus*. Naturwissenschaften.

[33] Kovac H., Stabentheiner A., Brodschneider R. (2015). What do foraging wasps optimize in a variable environment, energy investment or body temperature?. J. Comp. Physiol. A.

[34] Kovac H., Stabentheiner A., Brodschneider R. (2018). Foraging strategy of wasps—Optimisation of intake rate or energetic efficiency. J. Exp. Biol..

[35] Stabentheiner A., Kovac H. (2014). Energetic optimisation of foraging honeybees: Flexible change of strategies in response to environmental challenges. PLoS ONE.

[36] Stabentheiner A., Kovac H. (2016). Honeybee economics: Optimisation of foraging in a variable world. Sci. Rep..

[37] Kovac H., Stabentheiner A. (2012). Does size matter–Thermoregulation of ‘heavyweight’ and ‘lightweight’ wasps (*Vespa crabro* and *Vespula* sp.). BIO.

[38] Kovac H., Stabentheiner A., Schmaranzer S. (2010). Thermoregulation of water foraging honeybees–Balancing of endothermic activity with radiative heat gain and functional requirements. J. Insect. Physiol..

[39] Käfer H., Kovac H., Simov N., Battisti A., Erregger B., Schmidt A.K.D., Stabentheiner A. (2020). Temperature tolerance and thermal environment of European seed bugs. Insects.

